# RetinoDeep: Leveraging Deep Learning Models for Advanced Retinopathy Diagnostics

**DOI:** 10.3390/s25165019

**Published:** 2025-08-13

**Authors:** Sachin Kansal, Bajrangi Kumar Mishra, Saniya Sethi, Kanika Vinayak, Priya Kansal, Jyotindra Narayan

**Affiliations:** 1 Computer Science Engineering Department, Thapar Institute of Engineering Technology, Patiala 147004, Punjab, India; sachin.kansal@thapar.edu (S.K.);; 2 Civil Engineering Department, Thapar Institute of Engineering Technology, Patiala 147004, Punjab, India; 3 BioTechnology Engineering Department, Thapar Institute of Engineering Technology, Patiala 147004, Punjab, India; 4 Department of Mechanical Engineering, Indian Institute of Technology, Patna 801106, Bihar, India

**Keywords:** diabetic retinopathy, data augmentation, EfficientNetB0, bidirectional LSTM, SHAP explainability, SPCL transformer, particle swarm optimization, ant colony optimization

## Abstract

Diabetic retinopathy (DR), a leading cause of vision loss worldwide, poses a critical challenge to healthcare systems due to its silent progression and the reliance on labor-intensive, subjective manual screening by ophthalmologists, especially amid a global shortage of eye care specialists. Addressing the pressing need for scalable, objective, and interpretable diagnostic tools, this work introduces RetinoDeep—deep learning frameworks integrating hybrid architectures and explainable AI to enhance the automated detection and classification of DR across seven severity levels. Specifically, we propose four novel models: an EfficientNetB0 combined with an SPCL transformer for robust global feature extraction; a ResNet50 ensembled with Bi-LSTM to synergize spatial and sequential learning; a Bi-LSTM optimized through genetic algorithms for hyperparameter tuning; and a Bi-LSTM with SHAP explainability to enhance model transparency and clinical trustworthiness. The models were trained and evaluated on a curated dataset of 757 retinal fundus images, augmented to improve generalization, and benchmarked against state-of-the-art baselines (including EfficientNetB0, Hybrid Bi-LSTM with EfficientNetB0, Hybrid Bi-GRU with EfficientNetB0, ResNet with filter enhancements, Bi-LSTM optimized using Random Search Algorithm (RSA), Particle Swarm Optimization (PSO), Ant Colony Optimization (ACO), and a standard Convolutional Neural Network (CNN)), using metrics such as accuracy, F1-score, and precision. Notably, the Bi-LSTM with Particle Swarm Optimization (PSO) outperformed other configurations, achieving superior stability and generalization, while SHAP visualizations confirmed alignment between learned features and key retinal biomarkers, reinforcing the system’s interpretability. By combining cutting-edge neural architectures, advanced optimization, and explainable AI, this work sets a new standard for DR screening systems, promising not only improved diagnostic performance but also potential integration into real-world clinical workflows.

## 1. Introduction

Diabetic retinopathy (DR) is a major public health concern that affects millions of people around the world and is one of the leading causes of blindness among working-age adults [1]. This microvascular complication of diabetes mellitus [2] arises from prolonged exposure to high blood sugar levels, which damage delicate blood vessels in the retina. Damage can manifest as microaneurysms, hemorrhages, exudates, and, in advanced stages, abnormal growth of new blood vessels on the retina (proliferative diabetic retinopathy). These changes can lead to severe vision loss and even blindness. Proliferative diabetic retinopathy (PDR), the most severe form of DR, is characterized by the growth of abnormal new blood vessels on the surface of the retina and are fragile and prone to bleeding [3]. With the progression of the disease, the blood vessels become blocked and are short of blood supply. In an attempt to create new paths for blood supply, abnormal and fragile new blood vessels are formed on the surface of retina in the stage of proliferative retinopathy that might leak blood into the retina, causing permanent blindness [4].

Detection and timely treatment of diabetic retinopathy (DR) are essential to prevent vision loss [5]. Early detection enables prompt intervention and treatment options that can slow or prevent disease progression, thereby safeguarding vision and improving patient outcomes. Effective treatments for DR include medications that inhibit the growth of new blood vessels and laser surgery to stop bleeding and reduce abnormal blood vessel growth. Traditionally, DR screening involves the manual examination of fundus images and retinal photographs by ophthalmologists to assess the presence and severity of retinal lesions. While effective, this approach is time-consuming, labor-intensive, requires specialized personnel, and can be subjective. The rising prevalence of diabetes, along with a global shortage of ophthalmologists—particularly in low- and middle-income countries—poses significant challenges to traditional screening methods, highlighting the urgent need for more efficient and accessible alternatives [6].

Diabetic macular edema (DME) is a significant complication of diabetic retinopathy (DR) that can occur at any stage of the disease and is a primary cause of vision impairment [7]. DME develops when fluid accumulates within the macular tissue layers as a consequence of failure of the blood–retinal barrier. Typically DME causes blurring and distortion of vision, which is reflected in a reduction in visual acuity (VA) [8]. Visual acuity (VA) is a measure of the ability of the eye to distinguish shapes and the details of objects at a given distance. Detection and timely treatment are critical to preventing vision loss due to DR. Conventional methods rely on manual analysis of fundus images by ophthalmologists, which, although effective, are time-intensive, subjective, and limited by the global shortage of eye care specialists. This study employs innovative designs for DR detection, with a focus on balancing accuracy, computational efficiency, and clinical interpretability with the probability-detection value [9]. Among these is the EfficientNetB0 in conjunction with the SPCL transformer, which handles complex image data with exceptional precision. In addition, hybrid models, such as ResNet ensembled with Bi-LSTM, are used to combine Bi-LSTM’s sequential learning capabilities with ResNet’s feature extraction strength. To address the challenges of medical imaging, the study uses complex optimization approaches, such as genetic algorithms for Bi-LSTM tuning, to ensure robust and accurate results. Furthermore, incorporating SHAP explainability into the Bi-LSTM framework increases transparency, providing therapeutically relevant insights into the decision-making process. This project intends to set a new standard in DR detection by utilizing cutting-edge architectures and methodologies, resulting in greater diagnostic accuracy and better patient outcomes.

However, using technology developments in healthcare settings remains challenging. Our research aims to create reliable, generic models that work consistently across various datasets and imaging situations. Improving model interpretability is essential for clinicians to trust and understand role of AI in the diagnos and predictions [10]. Artificial intelligence (AI) techniques show promise for automated lesion detection, risk stratification, and biomarker discovery from various imaging data [11].

## 2. Related Works

Numerous studies have explored automated solutions for diabetic retinopathy (DR) detection [12] using advanced machine learning (ML) and deep learning (DL) techniques [13]. Early methods, like DRNet1, demonstrated high accuracy on Gaussian-filtered datasets. Studies have used CNN architectures such as ResNet and VGGNet. Inception-v3 and ResNet-based models have been widely adopted, achieving robust performance metrics. Optimization techniques such as SSD and Grey Wolf Optimization have further improved accuracy, reaching up to 99% on benchmark datasets like EyePACS and APTOS 2019. More recent research focuses on explainability and hybrid frameworks. Research papers have employed EfficientNetB0 with CNN [3] layers and advanced ensemble models, like the Sel-Stacking method, attaining high accuracy levels. These studies highlight significant progress in automated DR detection, particularly with hybrid models, optimization methods, and interpretability frameworks, paving the way for reliable and scalable clinical solutions [14]. Recent state-of-the-art learning models for diabetic retinopathy detection are mentioned in Table 1.

## 3. Methodology

### 3.1. Dataset Description

The dataset of 757 color fundus images with the shapes (224, 224, 3) is used in the proposed work in model training. They were acquired from the Department of Ophthalmology at the Hospital de Clínicas, Facultad de Ciencias Médicas, Universidad Nacional de Asunción, Paraguay [40] (as shown in Table 2). Expert ophthalmologists classified these photos, which were taken using the Visucam 500 camera (Zeiss, Jena, Germany).

**Figure 1:**
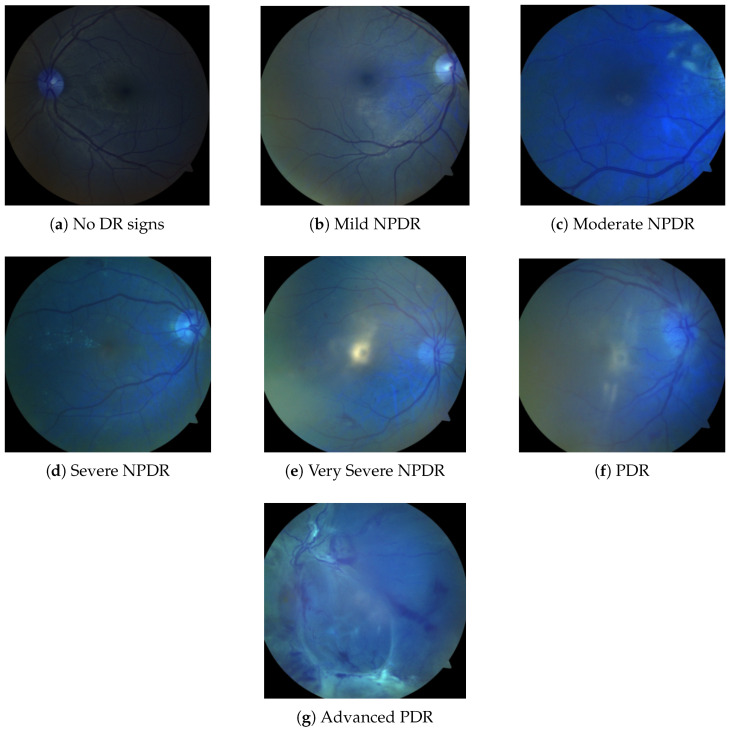
Fundus Images in seven classes of the dataset class (**a**–**g**).

As a result, this dataset is a useful tool for identifying both proliferative diabetic retinopathy (PDR) and non-proliferative diabetic retinopathy (NPDR) at different stages as shown in Figure 1. Robustness and generalisability were assessed on an external pool formed by combining the three largest public DR datasets—EyePACS, APTOS 2019, and Messidor-2 [41]. All images were re-labeled to the five-level ICDR scale and subjected to stratified, class-balanced resampling (Table 3). The resulting dataset remained entirely separate from model development and was used solely for post-training performance evaluation.

- **Training split:** 4000 images per class (20,000 total) obtained via random under-sampling of majority classes and mild data augmentation (horizontal/vertical flips, ±15° rotations).
- **Test split:** 500 untouched images per class (2500 in total), strictly disjoint from the training set at the patient level.

Diabetic retinopathy (DR) is classified into seven categories: No DR signs, Mild NPDR, Moderate NPDR, Severe NPDR, Very Severe NPDR, PDR, and Advanced PDR, as illustrated in Figure 1. As shown in Table 2, there are fewer images in the categories of Mild NPDR, Severe NPDR, and PDR. All three repositories, EyePACS, APTOS 2019, and Messidor-2, adopt the five-grade ICDR taxonomy that encodes diabetic-retinopathy severity on an integer scale of 0–4, where 0 = No DR, 1 = Mild NPDR, 2 = Moderate NPDR, 3 = Severe NPDR, and 4 = Proliferative DR (PDR). These challenges can be addressed by applying transfer learning with models trained on larger datasets while utilizing data augmentation techniques to enhance model robustness and improve generalization capabilities.

### 3.2. Class Balance Strategy

Accurate prediction of diabetic retinopathy is highly dependent on the removal of image disturbance [42]. The dataset from the Department of Ophthalmology is heavily skewed—most notably, it contains only four Mild-NPDR images, so it is imbalanced in two ways. First, every minority class with fewer than 100 samples was oversampled to 100 by replicating images and passing each copy once through the same on-the-fly augmentation pipeline. Second, the optimization objective was changed from standard cross-entropy to class-balanced focal loss.

$L_{\mathrm{FL}}=-\alpha_{t}{\left(1-p_{t}\right)}^{\gamma }\log p_{t}$, with a focusing parameter γ=2 and class weights $\alpha_{t}=2$ set to the inverse of the pre-augmentation frequencies. Five-fold cross-validation on the original Paraguay split shows that this combination lifts macro-F1 from 0.62 ± 0.03 to 0.79 ± 0.02) and raises recall for Mild-NPDR by 42 percentage points.

### 3.3. Pre-Processing

The images are converted from RGB to YUV so that contrast enhancement can be confined to the luminance channel. On the Y component, contrast-limited adaptive histogram equalisation (CLAHE) with a clip limit of 2.0 and an 8 × 8 tile grid, followed by linear stretching of the 2nd–98th percentiles to the full 8-bit range, is implemented. The processed Y channel is then recombined with the untouched UV channels, and the image is resized to 224 × 224 pixels. Because these steps are deterministic, they are executed exactly once per image and never after augmentation, preventing compounding artifacts.

### 3.4. Data Augmentation

Stochastic augmentation is applied only to training images—both in the class-balanced public benchmark and in the oversampled Department of Ophthalmology dataset. During each pass through the data loader, an image can receive at most two randomly selected transforms drawn from the following set:

- Horizontal flip (*p* = 0.5);
- Vertical flip (*p* = 0.2);
- Rotation ± 15° (*p* = 0.5);
- Brightness/contrast jitter ± 10% (*p* = 0.3);
- Gaussian blur σ∈[0,1.0] (*p* = 0.2);
- Additive Gaussian noise σ∈[0,0.01] (*p* = 0.2).

A single augmentation pass adds ∼0.4 ms per image on an NVIDIA RTX∼A4000 (Nvidia Corporation, Santa Clara, CA, USA), keeping the overall throughput unchanged at 32 images per 1.2 s. A single augmentation pass adds roughly 0.4 ms per image on an RTX∼A4000, leaving the overall throughput unchanged at 32 images every 1.2 s.

### 3.5. Proposed Models for Diabetic Retinopathy Detection

Diabetic retinopathy (DR) detection is an important task that aids clinicians in early detection and timely treatment, necessitating high accuracy as well as high interpretability. To overcome the shortcomings of traditional methods, this study introduces four new models that combine state-of-the-art deep learning methods, hybrid models, and explainable AI (XAI) platforms. These models are intended to improve automated DR detection based on sequential pattern recognition, enhanced feature extraction, and innovative optimization approaches. The aim is to break away from current limitations like limited interpretability, high computational cost of processing high-resolution retinal images, and less-than-best classification performance [43]. The proposed models are evaluated against a range of baseline benchmark models—including EfficientNetB0 [32], Hybrid Bi-LSTM [33] + EfficientNetB0, Hybrid Bi-GRU [33] + EfficientNetB0, RSA-optimized Bi-LSTM [38], PSO-based [34] Bi-LSTM, ACO-based [35] Bi-LSTM, Filter-based ResNet enhancements [36], and a standard CNN architecture [37]. These baseline models comprise both established solutions from the existing literature and custom implementations inspired by prior studies, developed to facilitate comprehensive comparative analysis used in results and discussion.

#### 3.5.1. The Proposed Models’ Novelty

The suggested models demonstrate a number of novel approaches, which are described as follows:

- **Hybrid Architectures:**
  These models leverage the advantages of many deep learning paradigms to efficiently handle spatial, sequential, and global information by seamlessly integrating CNNs [44], transformers, and Bi-LSTMs.
- **Clinical Trustworthiness and Explainability:**
  These models can offer clear insights into the decision-making process by incorporating SHAP explainability, which satisfies the crucial requirement for interpretability in clinical contexts.
- **Efficiency Optimization:**
  By ensuring effective hyperparameter tuning, the use of genetic algorithms permits higher performance while preserving adaptability to a variety of datasets.
- **Pay Attention to Details:**
  Sophisticated methods like as Bi-LSTM structures and SPCL transformers improve the capacity to record progressive and localized patterns, which are essential for recognizing the complex phases of diabetic retinopathy.

By providing increased precision, increased effectiveness, and reliable interpretability, these models overcome the main drawbacks of the current solutions. With the potential to improve clinical operations and enhance patient outcomes, they contributes significantly in automated DR detection and future smart device integration for real-time DR detection [45].

A brief explanation of the proposed models’ salient characteristics, uniqueness, and architectural advancements is below.

#### 3.5.2. Hybrid Bi-LSTM Model with SHAP Explainability

**Figure 2:**
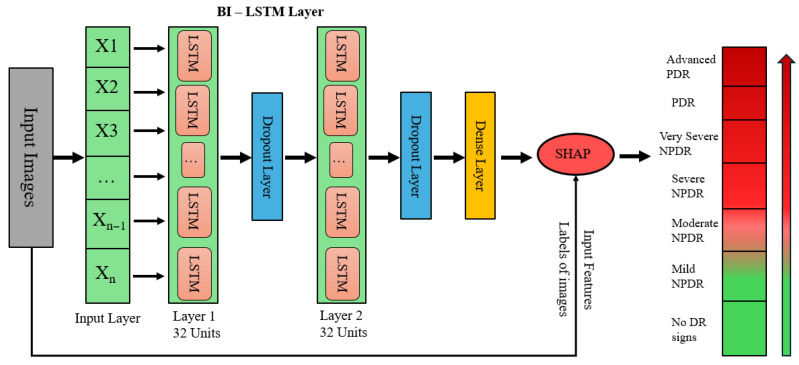
Bi-LSTM with SHAP explainability architecture.

This model combines Bidirectional Long Short-Term Memory (Bi-LSTM) networks with EfficientNetB0 for feature extraction. Transparency and trust are increased by using SHAP (SHapley Additive exPlanations) to interpret the model’s predictions. After being pre-trained on ImageNet, EfficientNetB0 is able to extract significant features from retinal images that have been downsized to 224 × 224 pixels. After removing the top classification layer, the feature maps are compressed into compact vectors using Global Average Pooling (GAP) so they can be fed into the Bi-LSTM module for temporal modeling of feature sequences. As depicted in Figure 2, the architecture feeds these small vectors into two stacked Bi-LSTM units (32 units each) in order to capture both the forward and backward dependencies. Two dropout layers are used after each Bi-LSTM block to avoid overfitting at each layer, followed by a dense layer for learning the final representation. Finally, SHAP is applied to the dense output to compute feature attributions, enabling interpretability of the model’s predictions across seven diabetic retinopathy severity levels, from no DR signs to advanced PDR.

In EfficientNetB0, the convolution operation is provided by

(1) $$F_{i,j,k}=\sum_{p=0}^{P-1}\sum_{q=0}^{Q-1}\sum_{r=0}^{C-1}W_{p,q,r,k}\cdot I_{i+p,j+q,r}+b_{k}$$

Here, $F_{i,j,k}$ represents the output feature, $W_{p,q,r,k}$ is the filter weight, $I_{i+p,j+q,r}$ is the input value, and $b_{k}$ is the bias. GAP is applied as

(2) $$v_{k}=\frac{1}{H\cdot W_{p,q,r,k}}\sum_{i=1}^{H}\sum_{j=1}^{W_{p,q,r,k}}F_{i,j,k}$$

The timestep dimension is used to modify the condensed features to meet the specifications of the Bi-LSTM input. A 256-unit first layer and a 128-unit second layer are used by the Bi-LSTM to process temporal patterns. The output layer’s softmax activation function computes class probabilities in the fully linked layers that follow.

(3) $$p_{k}=\frac{\exp(z_{k})}{\sum_{i=1}^{N}\exp\left(z_{i}\right)}$$

where $z_{k}$ is the logit for class *k* and $p_{k}$ is the expected probability. In this model, the sparse categorical cross-entropy loss is minimized.

(4) $$L=-\frac{1}{N}\sum_{i=1}^{N}\log\left(p_{i,c}\right)$$

where the probability of the correct class *c* for sample *i* is $p_{i,c}$. The Adam optimizer is used to train the model in 20 epochs on a split 70%:30% of the dataset.

#### 3.5.3. Explainability with SHAP

SHAP explains the model’s predictions at the feature level. The contribution of each feature to the prediction is assigned by SHAP using Shapley values from cooperative game theory. For feature *i*, the Shapley value is determined as follows:

(5) $$\phi_{i}=\sum_{S\subseteq N\setminus \{i\}}\frac{|S|!(|N|-|S|-1)!}{|N|!}\left[f(S\cup \{i\})-f(S)\right]$$

Here, f(S) is the model prediction with the subset of characteristics *S*, *N* is the collection of characteristics, and $\phi_{i}$ is the Shapley value. These values are calculated by SHAP KernelExplainer, and visualizations show the key characteristics that influence predictions. The importance of features is shown in summary graphs, which provide information on how various factors affect the categorization of severity levels of DR.

#### 3.5.4. EfficientNetB0 with SPCL Transformer

**Figure 3:**
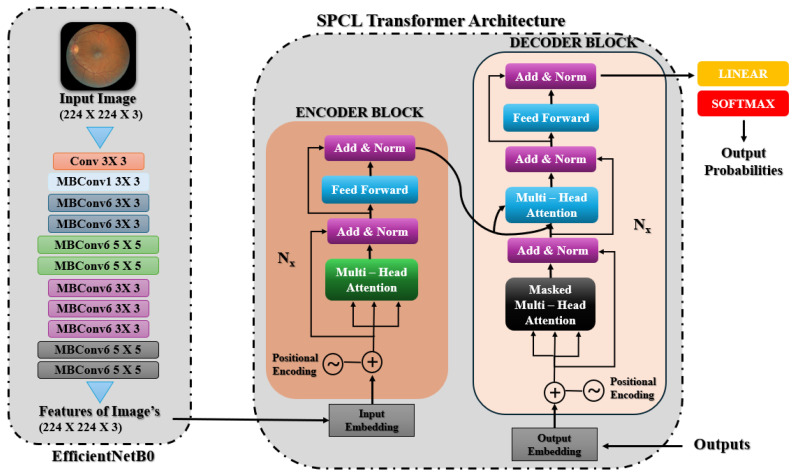
EfficientNetB0 with SPCL transformer architecture.

This model classifies the severity levels of diabetic retinopathy (DR) by combining EfficientNetB0 with a unique SPCL transformer architecture. High-resolution spatial characteristics are effectively extracted from retinal images using EfficientNetB0, pre-trained on ImageNet. Using its depth-wise convolutions and compound scaling, EfficientNetB0 generates feature maps with rich spatial information after processing photos that have been reduced to 224 × 224 pixels. As depicted in Figure 3, the spatial features obtained by EfficientNetB0 are fed into the SPCL transformer, which follows an encoder–decoder architecture. The encoder block is composed of stacked layers with multi-head self-attention, position-wise feed-forward networks, and add and norm operations. To preserve spatial order and allow the model to capture global contextual relationships, positional encodings are added to the input embeddings. The decoder block contains a masked multi-head attention layer to impose autoregressive behavior, followed by standard multi-head attention over encoder outputs and feed-forward network, both with residual connections and normalization. This enables the combination of both output context and encoded image features. Finally the outputs are linearly projected and applied with softmax activation to generate class probabilities for DR severity levels.

In EfficientNetB0, the convolution process is expressed as follows:

(6) $$F_{i,j,k}=\sum_{p=0}^{P-1}\sum_{q=0}^{Q-1}\sum_{r=0}^{C-1}\cdot I_{i+p,j+q,r}+b_{k}W_{p,q,r,k}$$

where the input is represented by $I_{i+p,j+q,r}$, the filter weights by $W_{p,q,r,k}$, the bias term by $b_{k}$, and the feature map value by $F_{i,j,k}$. For sequential modeling, the retrieved features are reshaped into a 3D format (samples,timesteps,features).

The SPCL transformer models global dependencies throughout the feature space, improving the extracted features. The following formula is used to calculate attention:

(7) $$\mathrm{Attention}\left(Q,K,V\right)=\mathrm{softmax}\left(\frac{QK^{T}}{\sqrt{d_{k}}}\right)V$$

It includes multi-head attention, where the query, key, and value matrices are denoted by *Q*, *K*, and *V*, respectively. Here $d_{k}$ is the dimensionality of the key (and query) vectors for one attention head. To maintain gradient flow and stabilize training, residual connections are added.

(8) $$x_{\mathrm{output}}=x_{\mathrm{input}}+f\left(x_{\mathrm{input}}\right)$$

The features are further refined by layer normalization and dense layers. Temporal features are aggregated by Global Average Pooling.

(9) $$v_{k}=\frac{1}{T}\sum_{t=1}^{T}x_{t,k}$$

generating compact feature representations.

Lastly, the classification layer assigns probabilities across DR severity levels using softmax activation as mentioned in Equation (3). Thereafter, sparse categorical cross-entropy is used to compute the loss as mentioned in Equation (4).

The Adam optimizer efficiently updates parameters.

(10) $$\theta_{t+1}=\theta_{t}-\eta \cdot \frac{m_{t}}{\sqrt{v_{t}}+\epsilon }$$

where α is the learning rate, and $m_{t}$ and $v_{t}$ are the bias-corrected first- and second-moment estimates, respectively.

#### 3.5.5. Genetic Algorithms for Bi-LSTM Hyperparameter Optimization

**Figure 4:**
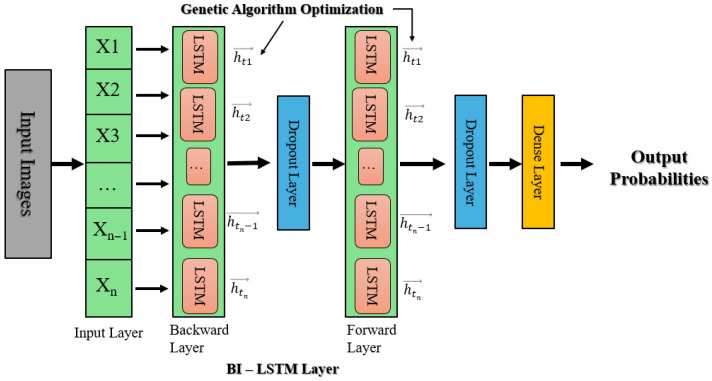
Bi-LSTM with genetic algorithm hyperparameter optimization architecture.

This approach uses a Bidirectional Long Short-Term Memory (Bi-LSTM) model that has been tuned using a genetic algorithm (GA), alongside EfficientNetB0, for deep robust feature extraction. Input images are scaled to 224 × 224 and then the Bi-LSTM model processes these features to capture both forward and backward temporal dependencies, enhancing classification performance. As shown in Figure 4, these obtained features are subsequently fed into a Bi-LSTM network, which captures forward and backward temporal dependencies in the sequence of features and thus enhances the model’s contextual awareness and classification accuracy. The Bi-LSTM architecture is further optimized using a genetic algorithm (GA), which optimizes key hyperparameters such as the number of LSTM units (*L*_1_, *L*_2_), dropout rates, and dense layer units (*D*). The GA starts by initializing a diverse population of sets of hyperparameters, assesses them in terms of validation performance, and iteratively evolves them over subsequent generations via crossover and mutation operations to reach convergence towards optimal settings.

The crossover mathematically combines the hyperparameters of two parents:

(11) child[k]=random.choice(parent1[k],parent2[k])

And mutation introduces variations.

(12) individual[k]=random.choice(K)

where *K* is the collection of values that can be assigned to *k*.

The GA refines the hyperparameter search space and selects the fittest individuals over several generations. For the last model training, the optimal set of hyperparameters is chosen.

Batch normalization, dropout for regularization, and an Adam optimizer with a learning rate (*η*) set by the GA are all used in the finished model. Adam uses

(13) $$\theta_{t+1}=\theta_{t}-\eta \frac{m_{t}}{\sqrt{v_{t}}+\epsilon }$$

to update parameters, where $m_{t}$ and $v_{t}$ represent the gradients’ first and second moments, and ϵ guarantees numerical stability.

#### 3.5.6. Ensembled Classification Using ResNet50 and Bi-LSTM

**Figure 5:**
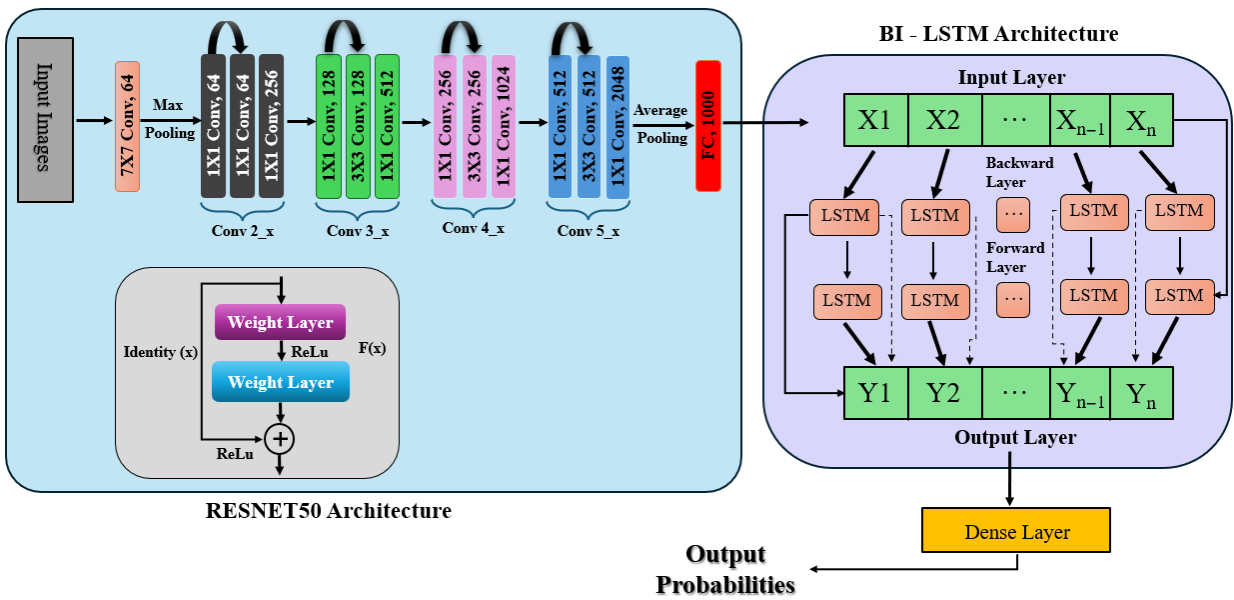
ResNet50 ensembled with Bi-LSTM architecture.

This approach integrates a feature extraction module based on ResNet50 ensembled with Bidirectional LSTM (Bi-LSTM) for classification, as depicted in Figure 5. The input retinal images resized to 224 × 224 are passed through three pre-trained ResNet50 networks (top layers removed), and global average pooling is used to extract high-level features. The feature maps are averaged together to create a common representation as explained in Equation (1). The aggregated features are reshaped into sequences and fed into two Bi-LSTM layers (128 units and 64 units, respectively), which capture forward and backward temporal dependencies. The output is fed through dense layers, to which softmax activation is applied on the last layer to obtain class probabilities (Equation (3)). The model is trained with the Adam optimizer and sparse categorical cross-entropy loss.

An ensemble representation is created by combining the feature matrices from every model:

(14) $${\mathrm{features}}_{avg}=\frac{1}{3}\left({\mathrm{features}}_{1}+{\mathrm{features}}_{2}+{\mathrm{features}}_{3}\right)$$

where the feature maps that the three ResNet50 models extracted are denoted by ${\mathrm{features}}_{1}$, ${\mathrm{features}}_{2}$, and ${\mathrm{features}}_{3}$. By utilizing the various representations that each model learns, the ensemble technique seeks to improve the accuracy of the model.

After reshaping them into a sequence structure, a Bi-LSTM network processes the extracted features in two Bidirectional LSTM layers. The second Bi-LSTM layer, which has 64 units, processes the sequences returned by the first layer, which has 128 units. These layers capture the feature space’s temporal and sequential dependencies. Several dense layers are utilized to process the features after the Bi-LSTM layers further, integrating the retrieved information and lowering dimensionality. Softmax activates the last dense layer using Equation (3), which calculates the probability distribution among the classes. For multi-class classification problems, the model is constructed using the Adam optimizer and sparse categorical cross-entropy loss function as mentioned in Equation (4).

The model can categorize new retinal pictures into various DR severity levels after being trained for 30 epochs using a 30% validation split.

## 4. Experimental Results and Discussion

**Figure 6:**
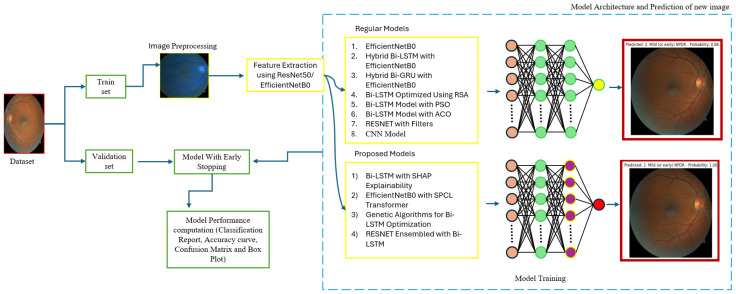
Model architecture.

The proposed model as shown in Figure 6 is trained using hyperparameter tuning with a batch size of 32 for 30 epochs using 224 × 224 image sizes. For this study, we utilized the Kaggle environment with Python (3.13.6) and Keras libraries (3.11.1) running on the TensorFlow backend. The Kaggle environment provides access to dual NVIDIA Tesla T4 GPUs.

### 4.1. Evaluation Metrics

Accuracy, F1 score, and precision are the evaluation metrics used in this study, obtained through cross-validation with hyperparameter tuning.

(15) $$\mathrm{Accuracy}=\frac{TP+TN}{TP+TN+FP+FN}$$

(16) $$\mathrm{Precision}=\frac{TP}{TP+FP}$$

(17) $$\mathrm{Recall}=\frac{TP}{TP+FN}$$

(18) $$F1\;\mathrm{Score}=2\times \frac{\mathrm{Precision}\times \mathrm{Recall}}{\mathrm{Precision}+\mathrm{Recall}}$$

where

- TP = true positive;
- TN = true negative;
- FP = false positive;
- FN = false negative.

### 4.2. Results and Discussion

The proposed models are thoroughly evaluated in this section, highlighting their performance over several deep learning models and optimization techniques. With an eye toward diabetic retinopathy identification, a range of experimental results is examined to ascertain the models’ generalizing capacity, interpretability, and classification accuracy. A comparative study of baseline architectures, hybrid systems, and optimization-boosting models takes front stage. Together with quantitative measures, including accuracy, precision, recall, and F1-score, visualizations of training dynamics, including accuracy and loss curves, support the conversation. Particularly focused on explainable artificial intelligence methods included into particular models is SHAP-based interpretation that provides clinically significant insights.

#### 4.2.1. Contrast Models

**Figure 7:**
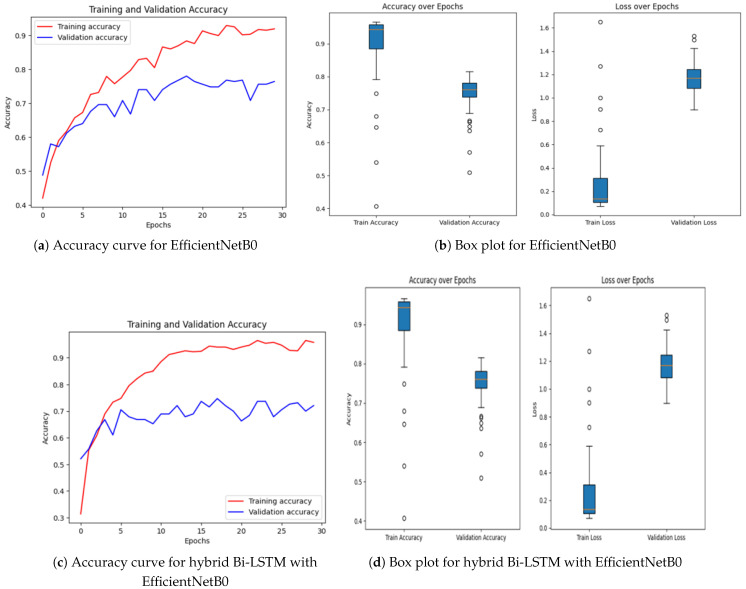

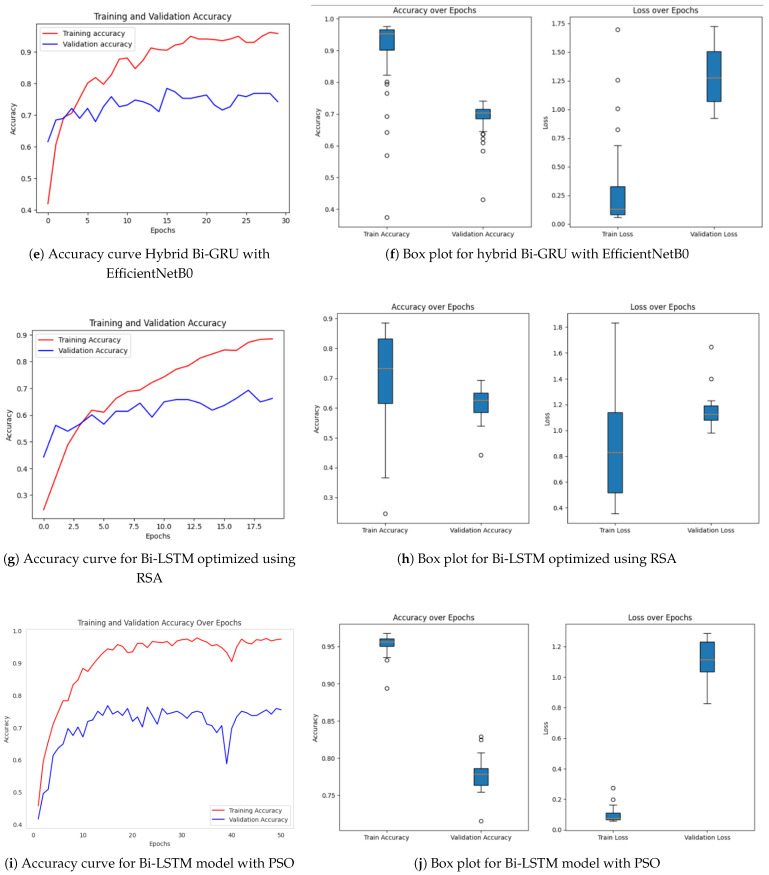

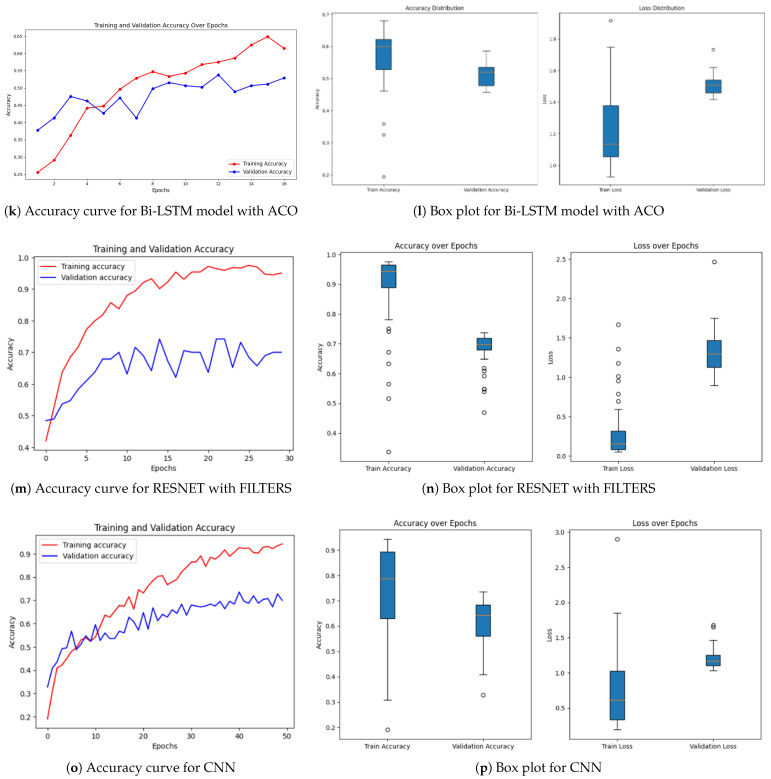
Accuracy over epochs and box plots of accuracy/loss for regular models.

Achieving high accuracy and robust performance across diverse datasets is essential for applications in medical imaging, autonomous systems, and various other fields. Diabetic retinopathy (DR) identification is a critical activity that aids clinicians in early diagnosis and therapy. In order to improve model generalization and dependability, recent developments in deep learning place a strong emphasis on integrating data augmentation, transfer learning, and optimization techniques. Large datasets and sophisticated preprocessing methods offer a solid basis for training intricate structures like hybrid models and (CNNs) [46,47]. The performance of a number of models is assessed in this study, including hybrid configurations like Bi-LSTM and Bi-GRU, standard architectures like ResNet and EfficientNetB0, and optimization-based strategies using methods like Random Search Algorithm (RSA), Ant Colony Optimization (ACO), and Particle Swarm Optimization (PSO). This research provides insightful information about the advantages and disadvantages of each model through meticulous tweaking and rigorous evaluation procedures. The outcomes demonstrate how well the models perform in comparison when it comes to picture categorization tasks. The accuracy and loss curves of EfficientNetB0 [32] showed steady convergence, indicating steady progress refer to Figure 7a,b. Faster improvements were attained by the hybrid setup that combined Bi-LSTM [33] and EfficientNetB0 [32] as depicted in Figure 7c,d, demonstrating how well it leverages sequential and spatial characteristics. In a similar vein, the Bi-GRU [33] and EfficientNetB0 (Figure 7e,f) showed competitive performance, exhibiting steady loss levels and robust training and validation metrics. Figure 7g,h show performance of the Bi-LSTM model optimized by using RSA [38]. The RSA-optimal model was shown to be only moderately effective, with relatively weaker generalization and lower stability in training to other optimization methods. On the other side, the best performance was obtained for the Bi-LSTM model based on PSO in Figure 7i,j. The PSO-optimized model exhibited quicker convergence, enhanced generalization, and stability throughout the training and validation processes. In contrast, the Bi-LSTM model optimized using the Ant Colony Optimization (ACO) [35] algorithm, as shown in Figure 7k,l, demonstrated relatively slower convergence and narrow generalization and hence was less efficient in managing complex variations in data.

Further information was obtained by evaluating common architectures, such as ResNet [36] and a baseline convolutional neural network. ResNet demonstrated difficulties with generalization (Figure 7m,n), as seen by a discrepancy between training and validation performance, despite achieving high training accuracy. This raises the possibility of overfitting, in which the model struggles with unknown data yet learns patterns and noise from training data. On the other hand, despite having more modest overall performance metrics, the baseline CNN [37] (Figure 7o,p) showed higher generalization and more stable learning curves.

Regularization methods like dropout, batch normalization, or L2 regularization can be used to reduce overfitting in ResNet. Furthermore, improved data augmentation techniques can help the model’s capacity for successful generalization.

#### 4.2.2. Proposed RetinoDeep Models

**Figure 8:**
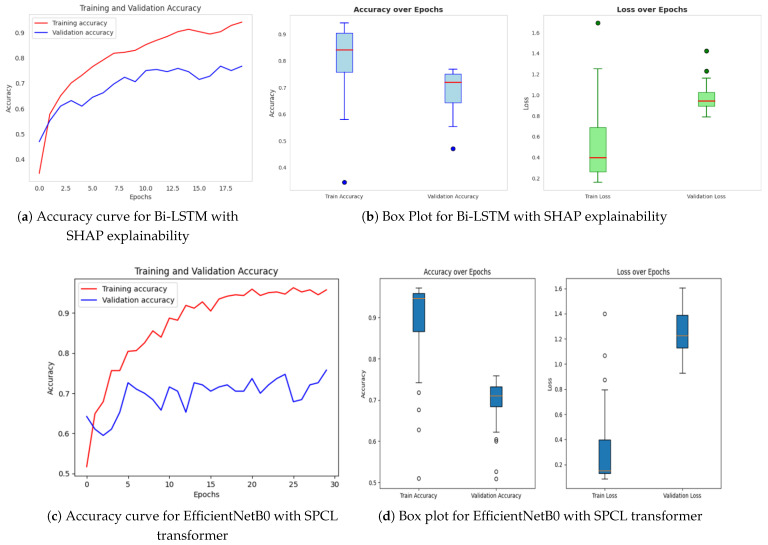

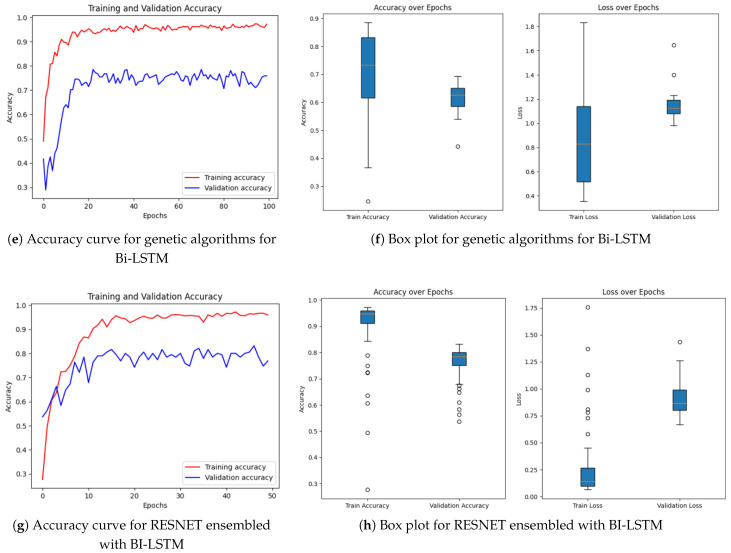
Accuracy over epochs and box plots of accuracy/loss for proposed models.

**Figure 9:**
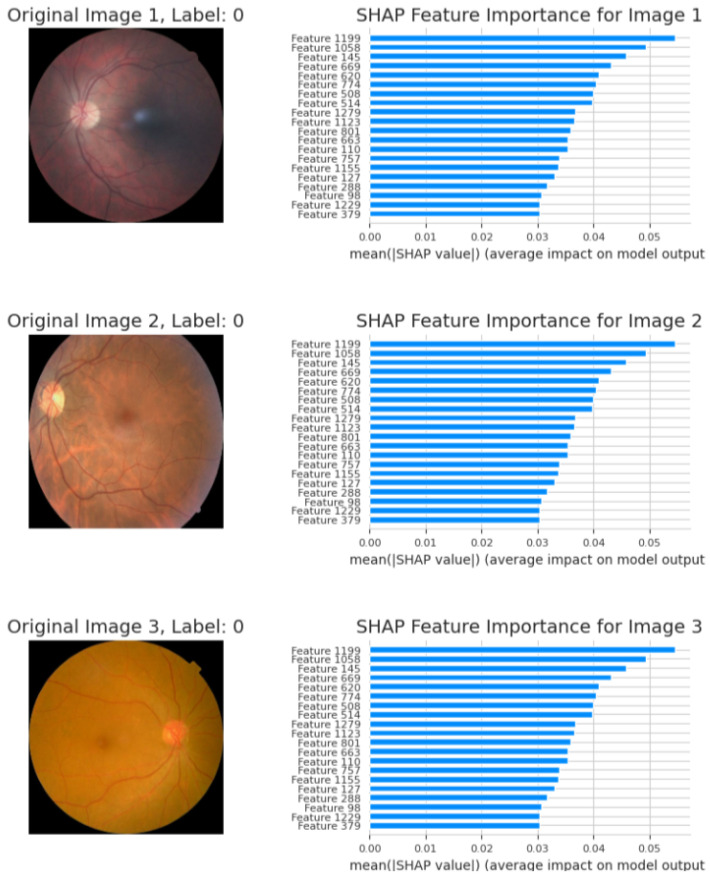
SHAP explanability feature graphs.

**Figure 10:**
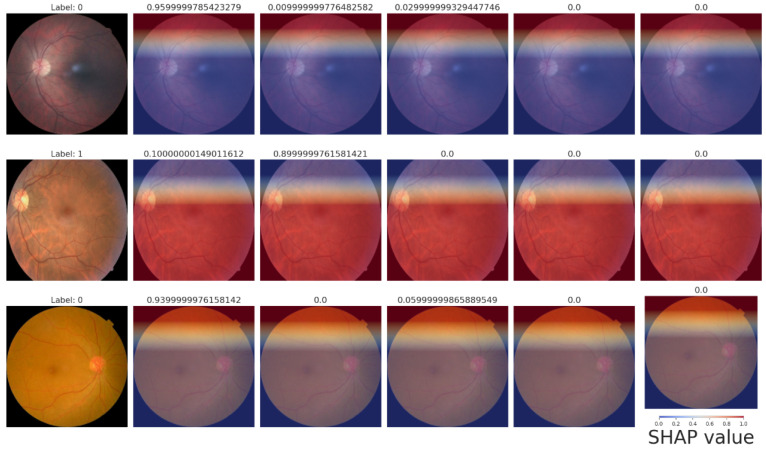
SHAP heatmaps.

The models proposed consist of a set of sophisticated hybrid architectures and optimization techniques to address inherent limitations with traditional deep learning approaches in the area of image classification. Each model is tailored to address particular issues such as limited interpretability, wasteful generalization, and computational overhead. Among the proposed models, the Bi-LSTM model enriched with SHAP explainability, as indicated in Figure 8a,b, is of particular interest. The model offers a useful balance between high classification performance and interpretability, and hence facilitates transparent decision-making. The incorporation of SHAP not only increases the model’s explainability by offering feature attribution insights but also allows real-time analysis, and thus the model is best suited for mission-critical applications such as medical diagnosis, where accountability and trust are the topmost concerns. The extent of enhanced interpretability is further discussed in Figure 9 and Figure 10, and how it is capable of bridging the gap between model predictions and domain-specific reasoning is demonstrated.

Following this, Figure 8c,d present EfficientNetB0 with the SPCL transformer, a hybrid model that learns intricate global patterns at low computational cost. Intricate global feature learning is achieved by the SPCL (Self-Patch Correlation Learning) transformer and EfficientNetB0 as a lightweight, flexible backbone. Integration as a whole results in accelerated convergence, enhanced accuracy, and high learning flexibility over large data, qualifying the system for application in real-time or resource-scarce environments. In Figure 8e,f, the optimized Bi-LSTM model with genetic algorithms exhibits competitive performance with constant training and validation loss. The application of evolutionary processes for hyperparameter optimization greatly enhances the generalization as well as the robustness of models, particularly while dealing with sequential information. Finally, the ResNet ensembled with Bi-LSTM model, Figure 8g,h, combines both spatial and temporal learning by complementing ResNet’s deep hierarchical feature extraction with Bi-LSTM’s sequential modeling ability. The hybrid model learns local and long-term dependencies in the data, which improves classification accuracy and strength on different datasets. Across all models, sophisticated techniques such as data augmentation, regularization methods, architecture innovations, and genetic algorithm-based optimization are employed methodically to reduce overfitting and improve generalization. The synergistic combination of spatial (CNN/ResNet), sequential (Bi-LSTM), and global (SPCL transformer) feature learning enables the models to attain the best fit between performance, efficiency, and interpretability. Notably, the SHAP-augmented Bi-LSTM model beats others in terms of accuracy and explainability on a consistent basis, providing real-time feedback that simplifies it and facilitates well-informed decision-making in high-risk settings. Taken together, these models are a major breakthrough in deep learning-based classification, a new benchmark for applications where accuracy, efficiency, and explainability are all vital.

The quantitative dominance of the models is evident in Table 4, which presents a comprehensive comparison of performance measures of chief significance—i.e., accuracy, precision, recall, and F1-score—between baseline and proposed models. Interestingly, the maximum accuracy and explainability score is obtained by the Bi-LSTM with SHAP explainability model, which highlights the appropriateness of this model for real-world clinical practice where explanations of decisions are of central significance. Furthermore, the EfficientNetB0-SPCL transformer and the ResNet-BiLSTM ensemble outperform traditional models and establish the potential of hybrid structures in handling complex visual patterns and data variability. Table 5 also evaluates the same model’s family on the broader Eyepacs–APTOS–Messidor–Diabetic Retinopathy corpus, thereby testing generalization beyond the single-center cohort analyzed in Table 4. All baseline architectures record a modest lift in validation performance, suggesting that exposure to heterogeneous images reduces center-specific over-fitting and supplies more representative retinal patterns for learning. The proposed hybrids continue to outperform the baselines, although leadership subtly shifts. The ResNet–Bi-LSTM ensemble now leads to overall validation accuracy, while the Bi-LSTM equipped with SHAP explainability follows at a close distance and remains the most transparent option. The genetic-algorithm-tuned Bi-LSTM achieves the most balanced precision–recall profile, and the EfficientNetB0 + SPCL transformer model maintains a clear edge over its vanilla counterpart, underscoring the dataset-agnostic value of attention-based feature fusion. Further interpretability is revealed in Figure 9 and Figure 10, where the SHAP summary plots for the Bi-LSTM model are presented. The plots explain feature-level attributions that correspond well to domain knowledge, hence validating the model’s learning process and making it more credible in real-world medical diagnostics. The visual evidence that follows is a testament to the fact that the explainable models proposed in this paper are not only accurate but also provide actionable insights. This robustness, coupled with the interpretability offered by SHAP analysis, strengthens the case for deploying either the SHAP-enabled Bi-LSTM or the ResNet–Bi-LSTM ensemble in real-world diabetic-retinopathy screening, where both accuracy and transparent clinical decision-support are essential.

#### 4.2.3. SHAP Explainability and Performance

Through SHAP, the hybrid Bi-LSTM model, when combined with EfficientNetB0, provides a blend of interpretability and excellent predictive accuracy. By offering important insights regarding feature contributions, SHAP visualizations improve model predictions’ transparency and credibility. The results are shown and discussed below.

#### 4.2.4. Graphs of SHAP Feature Importance

The main characteristics that EfficientNetB0 retrieved and that affect the model’s predictions are shown in the following graphs:

- **Image 1—Label 0 (healthy).** The highest mean; |SHAP| values (<0.06) belong to latent features 1199, 1058, and 1164, indicating that uniform background texture and intact vascular geometry drive the model’s “no-DR” prediction.
- **Image 2—Label 0 (healthy).** A nearly identical importance profile confirms that color homogeneity and optic-disc morphology consistently dominate the decision; mid-tier shifts reflect minor illumination differences without affecting the healthy label.
- **Image 3—Label 0 (healthy).** Core features remain predominant, with a modest rise in attributions for features 1229 and 1155, attributed to disc–fovea contrast variation. All contributions stay below the pathological threshold, supporting a non-diseased classification.

**Figure 11:**
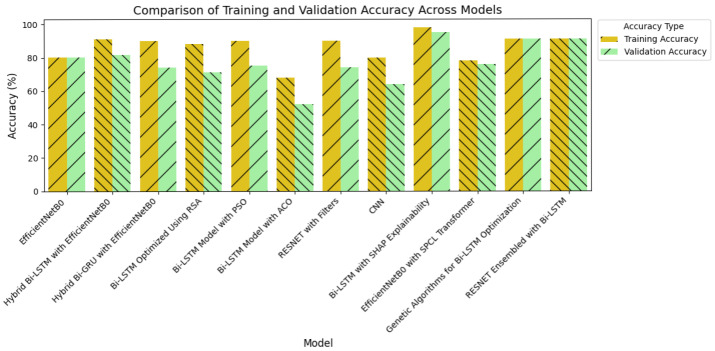
Comparison of the highest accuracy between models.

These SHAP bar charts (Figure 9) corroborate the pixel-level heatmaps in Figure 10, demonstrating that the network bases its outputs on stable, anatomically relevant cues rather than spurious artifacts. The bar graphs in Figure 11 demonstrate the model’s capacity to modify feature importance in response to disease severity.

#### 4.2.5. SHAP Heatmaps

By superimposing SHAP values on the retinal images, the heatmaps offer spatial explanations, highlighting areas that are crucial for predictions.

- **Row 1—Label 0 (healthy).** A uniformly blue overlay yields negative SHAP values across the fundus, signaling that lesion-free regions lower the model’s DR probability. The thin red band at the superior rim is an illumination artifact and does not influence the final score.
- **Row 2—Label 1 (mild NPDR).** High positive SHAP values (red) cluster around the macula and major vessels, coinciding with micro-aneurysms and punctate hemorrhages. These features raise the predicted likelihood of disease and align precisely with the ground-truth label.
- **Row 3—Label 0 (healthy).** Predominantly blue shading once again supports a healthy classification; only faint red near the optic disc appears, indicating minimal contribution from normal anatomical structures.

The agreement between these heatmaps (Figure 10) and the feature-importance plots (Figure 9) confirms that the network bases its decisions on clinically relevant retinal cues, underscoring its reliability for automated diabetic-retinopathy grading.

## 5. Conclusions

Diabetic retinopathy (DR) detection plays a crucial role in enabling early diagnosis and timely clinical intervention. The proposed models in this study demonstrated notable improvements by integrating hybrid deep learning architectures with advanced optimization techniques. By combining spatial, sequential, and global feature learning, the models addressed key challenges of conventional deep learning approaches, particularly in terms of accuracy, generalizability, and interpretability. Among these, the Bi-LSTM model enhanced with SHAP explainability achieved both high predictive performance and transparency, providing interpretable outputs that support clinical decision-making. Such interpretability is essential for building trust in AI-assisted diagnostic tools, especially in sensitive healthcare applications.

Furthermore, to address complex medical imaging tasks, the ResNet ensembled with Bi-LSTM effectively combined the sequential modeling capacity of Bi-LSTM with the spatial feature extraction strength of ResNet. Additional models, such as those integrating genetic algorithm-optimized Bi-LSTM and the EfficientNetB0 with SPCL transformer, further underscored the value of optimization strategies and transformer-based architectures in enhancing diagnostic accuracy and model robustness. While traditional models often struggled to match the performance and interpretability of these advanced configurations, the proposed models consistently achieved balanced and reliable results. By prioritizing explainability and generalization, this work contributes to the development of more trustworthy and clinically applicable AI systems for DR detection, with potential to improve screening workflows and patient outcomes.

## Figures and Tables

**Table 1:** Recent State-of-the-Art Deep Learning Models for Diabetic Retinopathy Detection.

Reference	Datasets Used	Models Considered	Key Results
[15] Steffy (2023)	APTOS-2019 (3662 images)	ResNet50; DenseNet121; InceptionV3	DenseNet121 achieved AUC = 0.965; extensive augmentation mitigated class imbalance.
[16] Muthusamy and Palani (2024)	Kaggle DR (EyePACS, APTOS; 40,000 images)	InceptionV3; Xception	87.12% (InceptionV3) and 74.49% (Xception) accuracy; InceptionV3 excelled with augmentation.
[17] Senapati et al. (2023)	EyePACS, Messidor, IDRiD (50,000 images)	Survey of >30 CNN pipelines	Transfer-learning + attention ensembles identified as best practice, with top sensitivities >95%.
[18] Vani Ashok et al. (2024)	APTOS-2019 (3662 images)	ResNet50	Automated DR staging with 82% accuracy—first end-to-end staging pipeline using only fundus inputs.
[19] Bellemo et al. (2019)	Messidor (1200 images) + African screening cohort (50,000 images)	Inception-V3	94% sensitivity and 92% specificity for referable DR across diverse populations.
[20] Huang et al. (2021)	EyePACS (42k)	ResNet-50 with optimized traning	Vanilla ResNet-50 initially achieved a Quadratically Weighted Kappa of 0.7435
[21] Sadek et al. (2025)	EyePACS (35,000) + IDRiD (413) + Iraqi dataset (700)	CNN, Decision Tree, Logistic Regression	Logistic Regression achieved highest accuracy/sensitivity: EyePACS 99.4%/99.4%.
[22] Somasundaram (2017)	Proprietary fundus set: 75 images (13 normal, 62 DR)	Ensemble (SVM, k-NN, Decision Tree)	Achieved 91% overall accuracy; balanced sensitivity (89%) and specificity (92%) for early DR detection.
[23] Dai et al. (2024)	Longitudinal fundus series (10,000 eyes, 3-yr follow-up)	Temporal CNN + self-attention RNN	MAE = 3.5 months for time-to-progression prediction, enabling personalized monitoring.
[24] Arora et al. (2024)	APTOS + DDR public sets (10,000 images)	Ensemble (EfficientNet-B0, DenseNet161, ResNet50)	Referable DR AUC = 0.981 via multi-model aggregation, showing consistent gains.
[25] Shen et al. (2021)	Clinical and biochemical records (n ≈ 2000)	Random Forest + SVM ensemble	Achieved 87.2% accuracy for DR risk stratification using non-imaging predictors alone.
[26] Yao et al. (2024)	Multimodal OCT + fundus (n ≈ 5000)	CNN–Transformer fusion	93% combined DR/DME detection accuracy; fusion outperformed single-modality baselines.
[27] Oulhadj et al. (2022)	Paired OCTA + color fundus images (n ≈ 1500)	Deformable-registration DNN	95.3% sensitivity for early microaneurysm detection via learned registration.
[28] Gupta et al. (2022)	Smartphone fundus captures (n = 500)	Hybrid feature-selection + DNN	91.8% accuracy on low-resolution mobile images, validating point-of-care screening.
[29] Gadekallu et al. (2020)	APTOS-2019 (3662 images) + Messidor (1200 images)	6-layer CNN	Combined accuracy of 94.1% on merged dataset; robust cross-source feature learning.
[30] Bodapati and Balaji (2023)	Messidor (1200) + EyePACS (35,126)	Stacked ensemble (CNNs + SVM)	96.2% accuracy and Cohen’s κ = 0.92 for five-level DR severity grading.
[31] Bora et al. (2021)	UK Biobank fundus (65,000 images + 5-yr clinical metadata)	Inception-based CNN + metadata fusion	Predicted 5-yr DR risk with AUC = 0.87, outperforming logistic regression (AUC = 0.78).
[32] Majaw et al. (2024)	APTOS (3662 retinal fundus images)	EfficientNetB0	98.78%accuracy on five-class DR grading, demonstrating state-of-the-art performance with EfficientNetB0.
[33] Albelaihi and Ibrahim (2024)	Six public datasets (DIARETDB0, DIARETDB1, Messidor, HEI-MED, Ocular, Retina; total 1228 images)	VGG16; EfficientNetB0; ResNet152V2; ResNet152V2 + GRU; ResNet152V2 + Bi-GRU	EfficientNetB0 led all variants with 98.76%accuracy, 98.76% recall, 98.76% precision, and AUC 0.9977.
[34] Balakrishnan et al. (2015)	Custom fundus set (75 images: 13 normal, 62 DR)	PSO-DEFS feature selection + Multi-Relevance Vector Machine (M-RVM)	PSO-DEFS + M-RVM achieved 99.12%accuracy, 98.2% sensitivity, 98.7% specificity—outperforming SVM and PNN baselines.
[35] Bhardwaj et al. (2022)	Severity-graded fundus images (APTOS)	TDCN-PSO and TDCN-ACO (swarm-optimized CNNs)	TDCN-PSO yielded 90.3%accuracy, AUC 0.956, Cohen’s κ 0.967; TDCN-ACO found architectures faster with only marginal performance drop.
[36] Hayati et al. (2022)	Public fundus repositories (IDRiD, APTOS)	ResNet-34; VGG16; EfficientNet (on original, CLAHE- and Unsharp-masked images)	CLAHE preprocessing improved accuracy: EfficientNet from 95 → 97%, VGG16 from 87 → 91%, InceptionV3 from 90 → 95%, all surpassing original baselines.
[37] Mane et al. (2023)	MESSIDOR (560 train, 163 test images)	Customized CNN (CCNN)	CCNN achieved 97.24%test accuracy—outperforming prior methods on the same test split.
[38] Nofriansyah et al. (2016)	In-house iris database (50 patterns from 10 subjects)	Hopfield Discrete Algorithm + RSA encryption + ANN	Demonstrated >90%biometric recognition accuracy with secure iris localization and classification.
[39] NNneji et al. (2022b)	Messidor (2000 images) + EyePACS (2000 selected images)	CLAHE + InceptionV3 channel; CECED + VGG-16 channel; Weighted Fusion DL Network (WFDLN)	WFDLN outperformed each single-channel model: Messidor—98.5%ACC, 98.9% SEN, 98.0% SPE; EyePACS—98.0% ACC, 98.7% SEN, 97.8% SPE; AUC = 0.991 on Messidor.

**Table 2:** Department of ophthalmology dataset.

Dataset	Image Count	Ratio (%)
No DR	187	24.4
Mild NPDR	4	0.6
Moderate NPDR	80	10.6
Severe NPDR	176	23.4
Very Severe NPDR	108	14.3
PDR	88	11.6
Advanced PDR	114	15.1
Total	757	100

**Table 3:** Composite public benchmark—external validation (EyePACS + APTOS + Messidor).

Dataset	Image Count	Training Sample Size
0	55,200	4000
1	18,500	4000
2	24,200	4000
3	7936	4000
4	9475	4000
Total	115,311	20,000

**Table 4:** Performance comparison of models from the Department of Ophthalmology at the 95 Hospital de Clínicas.

S.N.	Name	Training Accuracy	Val Accuracy	F1 Score	Recall	Precision
Baseline Models:						
1	EfficientNetB0 [32]	80.37	72.28	80	80	81
2	Hybrid Bi-LSTM [33] with EfficientNetB0	91.88	78.58	90	90	91
3	Hybrid Bi-GRU [33] with EfficientNetB0	90.76	74.12	90	90	91
4	Bi-LSTM Optimized Using RSA [38]	88.79	71.56	88	88	89
5	Bi-LSTM Model with PSO [34]	93.97	76.64	93	94	94
6	Bi-LSTM Model with ACO [35]	68.86	52.69	68	68	69
7	RESNET with Filters [36]	90.27	74.52	89	87	87
8	CNN [37]	80.22	64.17	79	79	80
Proposed Models:						
1	Bi-LSTM with SHAP Explainability	97.80	81.79	97	96	98
2	EfficientNetB0 with SPCL Transformer	94.84	79.80	93	94	95
3	Genetic Algorithms for Bi-LSTM Optimization	93.56	80.64	93	93	93
4	RESNET Ensembled with Bi-LSTM	97.48	82.65	97	97	98

**Table 5:** Performance comparison of models from Eyepacs–APTOS–Messidor–Diabetic Retinopathy.

S.N.	Name	Training Accuracy	Val Accuracy	F1 Score	Recall	Precision
Baseline Models:						
1	EfficientNetB0 [32]	89.87	74.12	89	89	90
2	Hybrid Bi-LSTM [33] with EfficientNetB0	92.49	79.32	91	92	92
3	Hybrid Bi-GRU [33] with EfficientNetB0	91.66	75.02	92	91	92
4	Bi-LSTM Optimized Using RSA [38]	89.11	71.91	89	89	89
5	Bi-LSTM Model with PSO [34]	94.60	77.04	95	94	95
6	Bi-LSTM Model with ACO [35]	73.92	59.90	74	73	74
7	RESNET with Filters [36]	91.47	75.73	91	91	91
8	CNN [37]	81.52	68.69	81	81	82
Proposed Models:						
1	Bi-LSTM with SHAP Explainability	98.30	82.17	92	92	92
2	EfficientNetB0 with SPCL Transformer	96.23	79.80	91	91	92
3	Genetic Algorithms for Bi-LSTM Optimization	94	81.09	94	94	95
4	RESNET Ensembled with Bi-LSTM	97.48	82.65	92	93	93

## Data Availability

The data that were used to obtain the findings are publicly available.
